# Fabrication and Characterization of Plasmonic Nanopores with Cavities in the Solid Support

**DOI:** 10.3390/s17061444

**Published:** 2017-06-20

**Authors:** Bita Malekian, Kunli Xiong, Gustav Emilsson, Jenny Andersson, Cecilia Fager, Eva Olsson, Elin M. Larsson-Langhammer, Andreas B. Dahlin

**Affiliations:** 1 Department of Chemistry and Chemical Engineering, Chalmers University of Technology, 41296 Gothenburg, Sweden; malekian@chalmers.se (B.M.); kunil@chalmers.se (K.X.); gusemi@chalmers.se (G.E.); 2 Insplorion AB, Sahlgrenska Science Park, Medicinaregatan 8A, 41390 Gothenburg, Sweden; jenny.andersson@insplorion.com (J.A.); elin.langhammer@insplorion.com (E.M.L.-L.); 3 Department of Physics, Chalmers University of Technology, 41296 Gothenburg, Sweden; cecilia.fager@chalmers.se (C.F.); eva.olsson@chalmers.se (E.O.)

**Keywords:** nanostructures, nanopores, plasmons, colloidal lithography, adsorption, sensors

## Abstract

Plasmonic nanostructures are widely used for various sensing applications by monitoring changes in refractive index through optical spectroscopy or as substrates for surface enhanced Raman spectroscopy. However, in most practical situations conventional surface plasmon resonance is preferred for biomolecular interaction analysis because of its high resolution in surface coverage and the simple single-material planar interface. Still, plasmonic nanostructures may find unique sensing applications, for instance when the nanoscale geometry itself is of interest. This calls for new methods to prepare nanoscale particles and cavities with controllable dimensions and curvature. In this work, we present two types of plasmonic nanopores where the solid support underneath a nanohole array has been etched, thereby creating cavities denoted as ‘nanowells’ or ‘nanocaves’ depending on the degree of anisotropy (dry or wet etch). The refractometric sensitivity is shown to be enhanced upon removing the solid support because of an increased probing volume and a shift of the asymmetric plasmonic field towards the liquid side of the finite gold film. Furthermore, the structures exhibit different spectral changes upon binding inside the cavities compared to the gold surface, which means that the structures can be used for location-specific detection. Other sensing applications are also suggested.

## 1. Introduction

Metallic nanostructures are widely used as optical sensors due to their ability to create high and strongly localized electromagnetic field enhancements. Over approximately the last 20 years, advances in nanofabrication techniques have provided strong synergetic effects with affinity-based refractometric detection or surface enhanced spectroscopies [1,2,3,4]. In particular, the use of single metallic nanoparticles as extremely miniaturized sensors has received much attention because of the possibility of resolving individual binding events and creating extremely high density arrays [5,6]. Nevertheless, as long as resolution in surface coverage is the important parameter rather than number of molecules it is hard for plasmonic nanostructures to compete with the established and standardized surface plasmon resonance (SPR) method [7]. Also, again in terms of surface coverage, which is the parameter that is directly related to analyte concentration at equilibrium, one can argue that the race for improved sensitivity and resolution for nanoplasmonic sensors still has not reached a point where SPR has been surpassed [8]. Yet, nanostructures may provide other interesting benefits, which will never be achieved with planar surface techniques, such as the possibility to investigate how surface topography influences biomolecular binding. Plasmonic nanopores have been especially useful for this purpose by generating regions with negative curvature [9]. It is particularly interesting to liberate a volume underneath the metal film to create cavities with controlled curvature and dimensions.

However, fabrication of ‘deep’ nanopores or nanoscale cavities, i.e., holes that continue into the supporting solid material, remains relatively complicated and there are few examples in the literature. Bochenkov et al. prepared elevated nanopores by colloidal lithography simply by depositing an additional thin dielectric film before the metal film [10]. Unfortunately, the aspect ratio of the underlying cavity becomes very limited with this approach, i.e., the diameter is always much higher than the depth. In previous work, we introduced mask-on-metal colloidal lithography for dry etching of the solid support, thereby generating deeper cavities [11]. An inconvenience with this method is that it requires introducing a third material (Nb_2_O_5_) on the sensor surface and thus another vacuum deposition step. Further, using reactive ion etching (RIE) it is not possible to create an etch undercut to fully suspend the metal film [12]. One way to etch the underlying support in a more isotropic manner is by wet etching. Tabatabaei et al., showed that etching with HF can remove a glass support entirely to create a suspended gold film with nanopores [13]. This approach is promising but it has not been implemented on nanohole arrays to make individual cavities with controlled volumes.

In this work, we describe in detail how to fabricate two types of plasmonic nanopores. The first structure is referred to as ‘nanowells’, which are prepared by RIE as in previous work [11], but we here simplify the fabrication process such that no extra supporting thin film is needed and improve the sensitivity. The second structure is an entirely new type of nanopores we refer to as ‘nanocaves’, which are prepared by controlled wet etching. Both structures are easily fabricated over large areas by colloidal self-assembly and parallel processing steps. We image the structures by scanning electron microscopy (SEM) and atomic force microscopy (AFM). Focused ion beam (FIB) is used for cross section analysis of nanocaves and nanowells. The diameter and depth of the cavities (as well as the apertures) are shown to be highly homogenous and tunable. The refractive index sensitivities of the plasmonic resonances and characteristic shifts upon molecular binding are reported. The possibility to perform location-specific detection [9] by proper spectral analysis is evaluated. Finally, possible applications of these nanostructured sensors are discussed.

## 2. Materials and Methods 

Polystyrene-sulphate colloids (158 nm) were purchased from Microparticles GmbH, which we found to be the most reliable provider of monodisperse batches (3% size variation). Thiolated poly(ethylene glycol) (PEG) with monodisperse molecular weight of 2000 g/mol was purchased from Nanocs. NeutrAvidin was purchased from Thermo Fisher Scientific. Thiolated PEG was prepared in 0.9 M Na_2_SO_4_ at a concentration of 2 g/L. NeutrAvidin was prepared in phosphate buffered saline (PBS) at a concentration of 50 µg/mL. Extinction spectroscopy was performed using a fiber coupled tungsten lamp and photodiode array spectrometer (B&WTek). A flow cell for optical transmission from Insplorion AB was used. Extinction is presented as the natural logarithm of reference intensity (no flow cell) divided by measured intensity (after subtracting the dark counts in the detector). The liquid cell used only gives a minor offset in the extinction. SEM was performed with a Zeiss Supra and a TESCAN GAIA3 for FIB cross section. AFM measurement of nanocaves were performed using a Ntegra Prima (NT-MDT) with TiN coated NSG30 cantilevers.

## 3. Results

### 3.1. Fabrication of Nanostructures

Although many of the fabrication steps have been described in previous work [11,14,15,16], we still provide a complete description here for the convenience of readers who wish to reproduce the process using a single complete protocol. We also try to include several tips and tricks that increase the chance of success.

**Figure 1:**
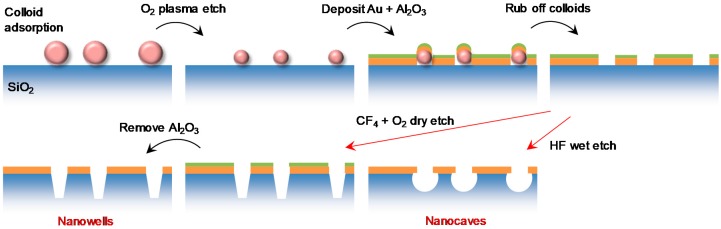
Fabrication steps for preparing ‘nanowells’ and ‘nanocaves’. Colloidal lithography is performed as in previous studies to make nanohole arrays. After removing the colloids, two different etch processes are used to produce the different structures. Anisotropic dry etching creates nanowells while more isotropic wet etching creates nanocaves. The Al_2_O_3_ layer (green) is removed in the HF etch process, but it does not influence Au (orange).

The fabrication process for preparing nanowells and nanocaves is outlined in Figure 1, starting with a clean fused silica (amorphous SiO_2_) support. For nanocaves, almost any type of ordinary glass may be used instead, while for nanowells we could only get the process to work well on fused silica. We denote the solid support as SiO_2_ throughout this paper. Colloidal self-assembly is first used to prepare a short-range ordered monolayer of monodisperse polystyrene particles [14]. When performing colloidal lithography, in order to avoid defects, it is important to keep the surface wet until the colloids can be fixed to the SiO_2_ support by heating [17], normally by pouring ~150 °C ethylene glycol onto the surface. Oxygen plasma was used to modify the diameter of the adsorbed particles, thereby defining the diameter of the final pores [16]. We observed that a relatively high pressure (250 mTorr) and low power (50 W) in the O_2_ plasma was beneficial for preserving the colloidal particle shape during shrinking, which may not always be straightforward [18]. Note that by small modifications in the colloidal lithography step it is also possible to prepare hexagonally long-range ordered arrays [19]. However, it is important to note that the pattern from the colloidal lithography process used here does not give a fully random pattern since there is a characteristic distance between neighboring particles [15,16,20] (here ~300 nm). No pores are connected to each other when the lithography is performed correctly.

After creating the colloidal pattern gold and alumina was deposited by physical vapor deposition using electron gun heating. A 1 nm Cr layer is necessary to provide adhesion between Au and SiO_2_. Ti also provides adhesion, but makes the samples sensitive to cleaning by H_2_O_2_. The role of the Al_2_O_3_ is to protect the gold when removing the colloids by rubbing the surface in liquid [16] and during RIE [11]. For all structures investigated in this work, we deposited 30 nm Au, but this thickness can naturally be changed. We found that in order to protect Au, at least ~15 nm Al_2_O_3_ is needed (we typically used 20 nm). Note that the thickness of the Au and Al_2_O_3_ layers limits the diameter of the final apertures since it must be possible to remove the colloids after depositing these layers. As a rule of thumb, if the sum of the Au and Al_2_O_3_ thickness is equal to the diameter, it is not possible to remove the colloids, but they can still be removed even if it is higher than the radius. To remove colloids, the surface needs to be kept in liquid (water works fine) and rubbed by something soft (such as a finger covered by a glove). Note that removal by tape stripping [14] tends not to work after O_2_ plasma treatment because the colloids become stuck very hard to the surface. The tape also leaves residues on the surface.

For preparing nanowells, the underlying material is anisotropically etched using RIE with CF_4_ (or NF_3_, CHF_3_, etc.) mixed with O_2_. The ratio of O_2_ to CF_4_ was 1:4 and the pressure was kept to a minimum (15 mTorr). For SiO_2_ this process requires only a low power (50 W) to achieve a relatively high etch rate (~20 nm/min depending on machine). After RIE the Al_2_O_3_ needs to be removed to expose the planar top Au film, which can be done by any weakly basic solution [11].

For preparing nanocaves, isotropic wet etching with HF was used to make a void, shaped roughly as a half sphere, in the underlying support. This transforms each aperture into a pore in a free-standing Au film. The samples were dipped in the etchant bath and rinsed thoroughly with water afterwards. We recommend caution since HF etchants are extremely toxic and exposure to skin can be fatal. The etch rate was found to be fast (~160 nm/min for 6% HF concentration). This process also removes the Al_2_O_3_ but does not influence Au [21]. Prolonged etching made the entire Au film peel off from the surface since the caves start to connect underneath the metal, i.e., the whole SiO_2_ support is lost evetually. Both the dry and wet etch rates were estimated based on measuring the depth for three different etch times, indicating a linear relationship.

An optional last step in the fabrication is to do thermal annealing to increase the stability of the samples, which will otherwise undergo recrystallization and faceting in Au with time. The temperature needs to be kept around 250 °C for recrystallization to occur reasonably fast (<1 h). Higher temperatures may alter the nanostructure due to mobility of Au atoms and the intrinsic stress at high curvature regions [22]. However, we noted that the standard cleaning procedure we used for the samples (H_2_O_2_ and NH_3_ in water at 80 °C) gives a similar recrystallization effect (and removes Al_2_O_3_), hence the cleaning may replace the annealing.

### 3.2. Electron Microscopy Imaging

**Figure 2:**
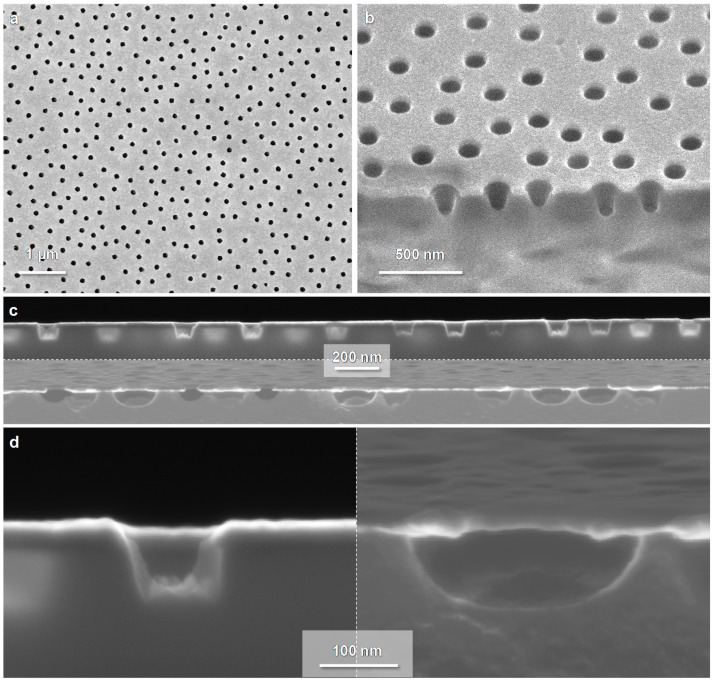
Electron microscopy imaging of the nanostructures. (**a**) Nanocaves imaged from above; (**b**) Nanowells imaged with tilt at a location for FIB analysis; (**c**) Sideview of cut samples (nanowells in top and nanocaves in bottom image); (**d**) Closeups showing the cross-section geometry of a nanowell (left) and a nanocave (right). Note that these images are just examples, i.e., different samples are shown so the diameter and depths are varying.

Representative SEM images of nanowells and nanocaves are shown in Figure 2. The structures appeared identical when imaged from above except that for nanowells it was possible to see parts of the walls in the underlying cavities. Also, the RIE causes a small increase in the average diameter, typically 10 nm after 5 min etching. This is expected since the RIE process cannot entirely avoid reactions with the exposed gold sidewalls, i.e., it is not completely anisotropic with respect to the vertical direction.

Since these nanostructures should be particularly useful for studying phenomena associated with nanoscale geometry [9] it is important to visualize the shape of the cavities in SiO_2_ underneath Au. A combined focused ion beam (FIB) and SEM was used to perform cross-sections (Figure 2b) and thus reveal the structure of the cavities (Figure 2c). For nanowells, the walls in SiO_2_ are clearly almost vertical but with a small inclination on the wall. It appears to be widely believed that the RIE process used can result in an undercut for an appropriate amount of O_2_ in the reactant gases. However, we always observed walls with a slight inclination towards the pore center even when making changes in the RIE recipe. In other words, the bottom of the wells were slightly smaller in diameter than the top aperture regardless of gas ratio, pressure, power, and machine used. The maximum depth of the nanowells prepared in this work was 200 nm but deeper wells up to at least 1 µm can most likely be achieved [18].

For nanocaves, the cross-section images can be used to analyze whether the wet etch is fully isotropic. Since the exposed SiO_2_ surface is a circle at the start of the etch and not a point source, the cavity is not expected to be a perfect half sphere. A fully isotropic etch would give a depth that is equal to the etch undercut along the Au film. This is not the case as shown in Figure 2d (the depth is ~100 nm and the undercut is ~50 nm). This is important to take into account since it influences how large cavities that can be prepared for the case of nanowells. When the caves start to connect laterally underneath the Au film the structure collapses. For the same reason, the maximum possible depth also depends on the diameter in Au. If the aperture is smaller, there is more SiO_2_ available to be etched laterally before the caves connect. The ‘not fully isotropic’ etch provides cavities that are actually very close to half spherical voids (Figure 2d) because of the size of the exposed initial SiO_2_ area.

### 3.3. Atomic Force Microscopy of Nanocaves

**Figure 3:**
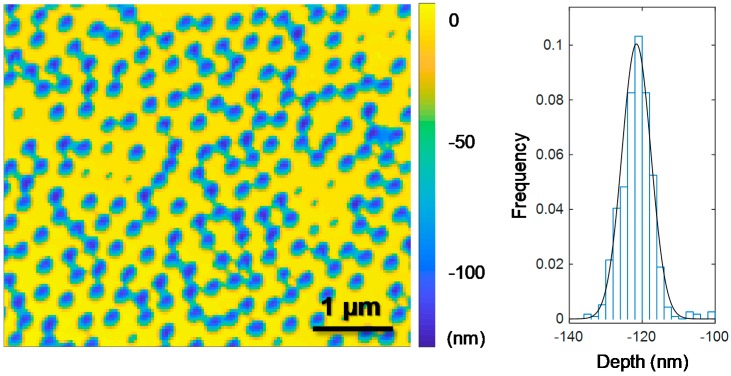
Atomic force microscopy of nanocaves. The Au film has been removed such that only the cavities in SiO_2_ are visualized. The sample was imaged by contact mode in air. On this sample, a small fraction of the wells have a much lower depth (excluded from the histogram) which is because a fraction of the colloids were not removed before HF etching. This occurs when approaching the limit of smallest possible diameter. Here the colloids were shrunk to 60 nm.

To acquire a more accurate measure for the depth of the nanocaves, we performed AFM in air after removing the metal film. This can be done by a commercial Au etch (I_2_/KI) or by applying a high positive electrochemical potential in a simple cell [22]. Figure 3 shows AFM measurements in air of (60 nm diameter) nanocaves that were prepared by etching in HF for 45 s. The histogram shows that the depth of the cavities in SiO_2_ is 120 nm with very little variation from cave to cave. It can also be seen that, for this etch time, neighboring caves are just starting to connect under the Au film which sets a limit of possible nanocave depths slightly above 100 nm in this case.

AFM measurements on nanowells only revealed a triangular line profile because the tip could not reach the bottom of such high aspect ratio cavities as long as they were above ~50 nm deep (not shown).

### 3.4. Extinction Spectra

**Figure 4:**
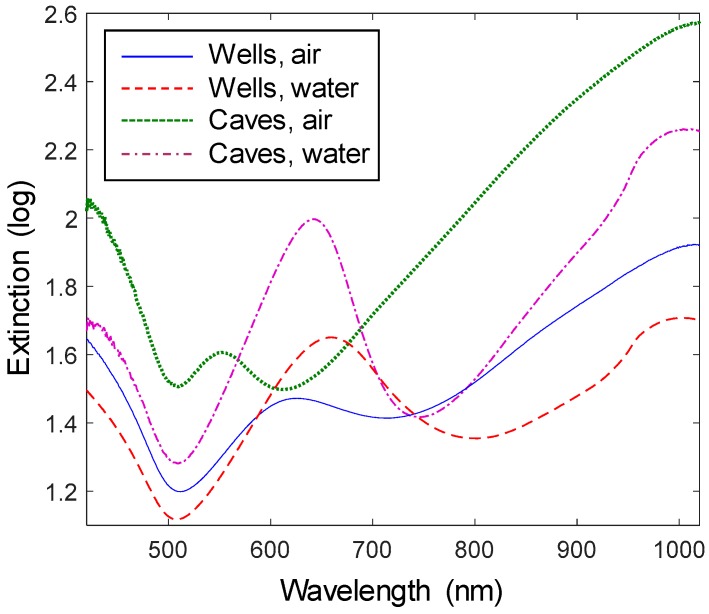
Extinction spectra of nanowells and nanocaves in air and water. The nanowells were 150 nm deep and 120 nm in diameter. The nanocaves were 70 nm in diameter and 100 nm deep. The small increase in extinction at 980 nm in water is due to its absorption band.

The extinction spectra (absorption + scattering) of the nanopores showed the characteristic asymmetric resonance (a ‘peak‘ and a ‘dip‘) associated with this kind of arrays of apertures in thin Au films [8] (Figure 4). Since we have characterized the plasmon modes extensively in previous work [11,15,20], no detailed description will be presented here. In brief, the short-range ordering of the apertures gives them similar properties to long-range ordered arrays and the peak represents Bloch wave coupling to surface plasmon modes with symmetric charge distribution across the finite Au film [20]. Because of this excitation, the resonance peak wavelength depends on the characteristic distance between the apertures (~300 nm for the colloids used in this work) and the thickness of the Au film. It does not, however, depend on the aperture diameter since that parameter is irrelevant for a grating-type coupling mechanism of light to plasmons [16]. For all spectra the peak is accompanied with an extinction minimum (transmission maximum) at nearby longer wavelengths, i.e., the dip. This dip behaves like a localized resonance in the sense that its field is more focused to the interior of the pore and its spectral position is sensitive to the shape of the apertures [16,20]. Here, we will focus on the effect on the optical properties when etching out the cavities in the solid support, i.e., how they compare with ‘regular nanohole arrays’ where the SiO_2_ support is not etched [14,16].

As the solid support is partly removed the effective refractive index (RI) of the environment decreases (as long as the cavities contain air). Indeed, in air the resonance peaks are quite blue shifted compared to ordinary nanohole arrays [16] and weak due to the interband transitions in Au which contribute to damping below ~600 nm. The nanocaves are more blue shifted than the nanowells, in agreement with more of the solid support being etched away. It should be kept in mind that the characteristic spacing between the apertures and the metal film thickness are kept constant. This means that the plasmon resonance condition only depends on the weighted average RI of the environment. The resonance peaks become much stronger in water and the red shift when going from air to water is higher for caves, again because of the higher cavity volume which makes the change in effective RI higher.

The sharpness of the resonances is also important for sensing applications. The nanocaves exhibit a significantly sharper resonance dip than the nanowells. This can be attributed to the differences in the etching processes. While the HF etch is essentially fully chemically selective to SiO_2_, the RIE with CF_4_ will attack the exposed Au walls to some extent, possibly by physical ‘milling’ rather than chemical etching. This is in agreement with the slight increase in diameter observed for nanowells (but not nanocaves) after etching. Such sidewall etching will make the shape of the apertures less controlled and as a result the extinction dip is red shifted and broadened.

### 3.5. Refractive Index Sensitivities

One motivation for etching the underlying support is to increase the refractometric sensitivity of nanoplasmonic sensors [10]. As more of the solid support is removed, a larger volume of liquid is present close to the metal where changes in RI due to molecular binding can occur, i.e., more of the probing volume is utilized. Therefore, increasing the cavity volume will increase the sensitivity defined as resonance spectral shift per RI change in the liquid. For nanowells, the increase will continue up to a certain point when the cavity bottom lies outside of the plasmonic field extension, which is typically on the order of ~50 nm for this thickness of Au [23]. However, for nanocaves the lateral etching under the Au film will still continue to liberate liquid volume that does have a strong plasmonic field. Note that the sensitivity is not expected to be influenced by the aperture diameter, although the degree of field confinement to the pores may be higher for smaller apertures [16].

**Figure 5:**
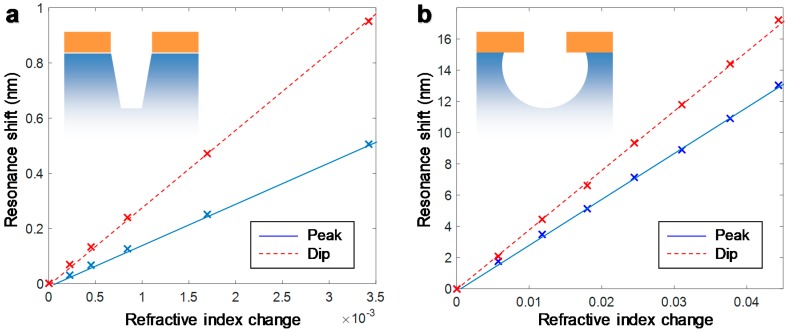
Refractive index sensitivities of (**a**) nanowells and (**b**) nanocaves. Note that a wider refractive index interval is analyzed for the nanocaves, but both structures exhibited linear sensitivities from pure water (1.33) up to at least 1.4. For these experiments the nanowells were 150 nm deep and 120 nm in diameter. The nanocaves were 70 nm in diameter and 100 nm deep.

We measured the sensitivity of the plasmon resonances using glycerol/water mixtures (Figure 5) for nanocaves that were 100 nm deep and nanowells that were also 100 nm deep. For nanowells, the peak sensitivity was 147 nm per RI unit, which is much higher than for our previous nanowell structures based on Nb_2_O_5_. The reason is that the plasmonic field is always focused to the side of the finite Au film where the RI is higher [15,20,24]. SiO_2_ does not differ too much in RI (1.48) from water (1.33) as compared to Nb_2_O_5_ (2.24) and, as a consequence, the field is strong on the metal-liquid interface, rather than the metal-support interface. This enhances the sensitivity and shows that these new nanowells are generally better for sensing applications. In addition, SiO_2_ is a material which is easier to chemically modify (or at least more studied) than Nb_2_O_5_. The variation in the sensitivity between samples was typically ±5 nm/RI unit. Increasing the depth did not lead to a noticeable increase in sensitivity beyond ~100 nm.

For nanocaves, the peak sensitivity was 294 nm per RI unit, which is more than twice as high as for structures where the support is not etched [16]. As mentioned, this is in part because the more isotropic etch liberates volume underneath the Au film. However, since the sensitivity increases by more than a factor of two, it is clear that the increment does not scale linearly with the volume liberated. The liquid probing volume is definitely not twice as high after etching the support. We attribute this effect as well to the asymmetric field distribution across the metal for the bonding surface plasmon mode [15]. In this interpretation, etching the SiO_2_ does not only liberate liquid volume. As mentioned, it also makes the field strength higher on the top side of the metal film. In conclusion, the wet etch process presented in this work leads to quite a substantial sensitivity enhancement.

The extinction dip is known to have a near field distribution more focused to the interior of the holes [16,20]. Indeed, a high sensitivity of 278 nm per RI unit is observed for the dip of nanowells upon etching away the SiO_2_. As expected, the etch undercut for nanocaves gives an even higher dip sensitivity or 388 nm per RI unit. Recall again that the metal thickness and aperture arrangement is the same. Although these values are higher than the peak sensitivities one must also take into account other factors when deciding which parameter to use in refractometric sensing. For one thing, the dip is a more broadened spectral feature and as a result it has a higher noise level. The uncertainty when tracking the resonance wavelength of the peak is below 0.01 nm, but around 0.02 nm for the dip (temporal resolution of 200 ms). In addition, the field extension in relation to the thickness of any molecular layer on the surface strongly influences the magnitude of the signal induced [23]. For these structures, the field extension at the peak wavelength is mainly determined by the thickness of the Au film, which influences the bonding surface plasmon mode (a thinner film makes the field more focused to the metal). At the dip wavelength, the field is mainly localized to the pores [16], so for this parameter to give a high signal it is important that molecules do bind to the cavities or the Au walls.

### 3.6. Molecular Binding and Location-Specific Detection

**Figure 6:**
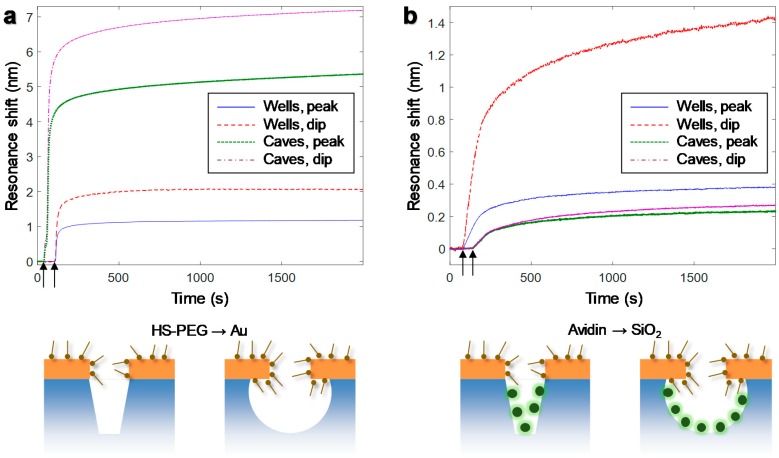
Real-time signals from material-specific molecular binding to the nanostructures. (**a**) Thiolated 2 kDa poly(ethyleneglycol) binding to Au. (**b**) Protein adsorption to SiO_2_. The temporal resolution is 250 ms and the data is not smoothened. The nanowells were 120 nm deep and 100 nm in diameter. The nanocaves were 70 nm in diameter and 100 nm deep.

We characterized the sensor response to molecular adsorption by first binding thiolated short (2 kDa) poly(ethylene glycol) chains to Au, followed by adsorption of the protein avidin inside the cavities. All binding was performed in PBS buffer and the data, i.e., centroid shift for both peak and dip [9], is presented in Figure 6. All binding was irreversible, i.e., the molecules remained on the surface (the signal did not decrease) upon rinsing. These material-specific reactions offer a way to not only evaluate the ability of the sensor to detect molecular binding events but also to provide information about the sensitivity distribution in the nanostructure. In the first step, molecules bind only to Au and in the latter only to SiO_2_ inside the cavities since the poly(ethylene glycol) prevents adsorption to Au. Further, by full spectral analysis it is possible to track both the peak and the dip shifts, thereby obtaining information about how the refractometric sensitivity is distributed in the nanostructures for the different resonance features.

It is clear from Figure 6a that the nanocaves provide higher signals for binding to Au, which makes them preferable from a performance point of view. This is expected when more of the Au surface is available for binding and when the field is higher on the top surface away from the solid support as explained above. For binding to SiO_2_, the nanocaves clearly give more moderate signals, even though they have a higher SiO_2_ surface area available. This can be explained by the fact that the SiO_2_ walls are not close to the pore where the sensitivity is highest. One should also keep in mind that most of the exposed area is Au and not SiO_2_ in both these structures. The nanowells give a reasonably high dip shift (~1.4 nm) upon protein adsorption inside the cavities. This illustrates that the ideal structure can depend on which material is chemically modified for binding a target. Note that, based on the size of the protein (~5 nm) and the cavities, the signal to noise is sufficient to detect one protein per cavity as long as it binds close to the metal [16].

Looking at the ratio of the signals in dip and peak for binding to Au and SiO_2_ it is clear that it is always higher than one and a higher value for this ratio indicates binding inside the cavities [9]. The ratio does not change so much for nanocaves when looking at the material specific binding (1.3 for Au vs. 1.2 for SiO_2_), while for nanowells the ratio is 1.8 for Au and 3.7 for SiO_2_. Although these ratios will vary somewhat depending on e.g., the depth of the cavities, it is clear that the nanowells give a higher change in the ratio when comparing binding to Au and the interior of the cavities. In other words, the considerable change in dip/peak signal ratio constitutes a built-in sensor signature for binding inside the cavities for the nanowells. This illustrates how full spectral analysis with multiple parameters can provide location-specific detection, at least in a qualitative manner.

## 4. Discussion

We have presented two types of plasmonic nanopores with etched solid supports and characterized them for refractometric sensing. In particular, we have described how the cavities formed influence the optical properties and the sensitivity compared to ordinary nanohole arrays [14,16,22]. The nanowells have good resolution for detecting binding to the interior of the nanopores and they give a clear spectral signature of preferential binding inside the cavities compared to the planar top surface. The nanocaves have the best refractometric sensitivity overall due to the undercut of the etch which liberates a larger liquid volume and increases the field strength at the top liquid interface. Both structures can be prepared over large areas by colloidal lithography (the largest we have prepared so far is 4 inch wafers). We found it particularly convenient to prepare the structures on large wafers and later cut them into small pieces to be used in experiments. The structures can also be cleaned and reused since they are robust enough to survive strong oxidizers [22].

One of the unique benefits of plasmonic nanostructures is the possibility to investigate phenomena associated with nanoscale geometry by utilizing the optical properties for non-invasive label-free readout. We have previously shown one example of such an application where virus particles bound to supported lipid membrane accumulate in cavities by multivalent interactions due to the surface curvature [9]. The improved nanowells presented here should be highly useful for such curvature-related applications. The curvature is then cylindrical in symmetry and increases towards the bottom of the cavities. For nanocaves, the negative surface curvature is instead more spherical.

Another suggested application is controllable immobilization of nanoscale objects to the cavities by material specific chemistry. The nanocaves have the additional benefit of giving a ’true’ pore structure in the sense that the solid support is completely removed just at the aperture. Also, the aperture diameter is less than the thickness of the film. This makes it possible to use the nanocaves as convenient model systems for investigating molecular translocation through solid state nanopores [25], a topic which remains intensely researched because of the possibility to perform DNA sequencing [26]. It should be particularly interesting to investigate how chemical modification of the gold influences molecular translocation, using the plasmonic signal for label-free real-time detection of transport. We also consider it intriguing to think about the possibility of entrapping molecules inside the cavities by actively controlling the translocation through the apertures.
